# Grain-size distribution dataset of supercritical flow sediments from a Gilbert-type delta that are associated with disaggregation bands

**DOI:** 10.1016/j.dib.2022.108792

**Published:** 2022-11-29

**Authors:** David C. Tanner, Christian Brandes, Jutta Winsemann

**Affiliations:** a Leibniz Institute for Applied Geophysics (LIAG), Stilleweg 2, 30655 Hannover, Germany; b Institut für Geologie, Leibniz Universität Hannover, Callinstr. 30, 30167 Hannover, Germany

**Keywords:** Disaggregation bands, Delta sediments, Supercritical flow deposits, Faults, Grain-size

## Abstract

This is a dataset of grain-size distribution in sub- and supercritical flow sediments of a Gilbert-type delta from an outcrop in North Germany. Thirteen samples of ca 2.5 kg were dried (at 105°C), and homogenised twice with a sample divider. A representative sample of 1-2 g was then analysed using laser diffraction. The grain-size distribution of the sand has a maximum between fine to medium sand, with a long fine fraction tail down to 0.06 µm and occasional coarse fractions (up to 1.5 mm) in some samples. Specific grain-size distributions correlate with the different sedimentary bedforms from which the samples were taken.

This data is important for two reasons: Firstly, sedimentary structures formed by Froude supercritical flows are controlled by grain-size. However, few studies have provided grain-size datasets from the natural record, which often have a much wider grain-size distribution than experimentally-produced supercritical flow deposits. Secondly, the sands were deformed subsequently by disaggregation bands, a type of geological fault that only develops in porous granular materials, i.e. well-sorted, medium sand. The disaggregation bands are indicative of seismic or even aseismic, creeping movement of basement faults.

**Specifications Table**

**Table:** 

Subject	Geology
Specific subject area	Grain-size distribution in supercritical flow sediments
Type of data	Table Graph Figure
How the data were acquired	Thirteen samples of sand were removed with a trowel and placed in plastic bags. The sedimentary structures were described and the location was photographed and GPS coordinates were noted. The sand samples, ca. 2.5 kg, were dried (at 105°C), and then homogenised twice using a sample divider (Retsch PT-100, with funnel and vibrating channel DR 100) to achieve representative sampling. Between 1.1 g and 2.0 g of sample were taken for the grain-size measurement. The actual amount of material is unimportant, the obscuration of the sample for the laser measurement being more important. Samples were placed in a test tube with 6.5 mL of ammonium hydroxide solution. The tubes were then spun for at least 12 hours to achieve true dispersion of the particles in the solution. Finally, the sample was measured using using a laser diffractometer (Beckman Coulter LS13320), consistent with ISO13320 (2009). Each measurement took 17 minutes; five grain-size profiles were measured, each taking 90 seconds. In addition, three samples were remeasured to test for replicability. These samples were found to be within 2.5% of the first samples and therefore the replicability was good. The data were exported as Excel tables and grain-size graphs in PDF format, which were then collated and plotted using Adobe Illustrator.
Data format	Raw Analyzed
Description of data collection	The five grain-size distribution profiles from each sample measurement were averaged, on the condition that the standard deviation was below 1% of the measurement at any one point on the curve.
Data source location	*City/Town/Region:* Freden, Lower Saxony *Country:* Germany *Latitude and longitude for collected samples:* N51°56′08.6″ – N51°56′13.2″ E9°51′50.8″ – E9°51′43.9°
Data accessibility	Repository name: **figshare** Data identification numbers: Figure 1: https://doi.org/10.6084/m9.figshare.21581805.v1 Figure 2: https://doi.org/10.6084/m9.figshare.20749165.v2 Figure 3: https://doi.org/10.6084/m9.figshare.20764768.v2 Figure 4: https://doi.org/10.6084/m9.figshare.20764804.v2 Tables 1, 2 and 3: https://doi.org/10.6084/m9.figshare.20764990.v1 Raw grainsize distribution curves for all samples, including tests for reproducibility https://doi.org/10.6084/m9.figshare.21317088.v1 Raw grainsize distribution curves as Excel tables (one sample per tab) https://doi.org/10.6084/m9.figshare.21317238.v1
Related research article	C. Brandes, D.C. Tanner, H. Fossen, M. Halisch, K. Müller, Disaggregation bands as an indicator for slow creep activity on blind faults. Communications Earth & Environment 3 (2022) 99. https://doi.org/10.1038/s43247-022-00423-8

## Value of the Data

The grain-size distribution data are of value to the scientific community because:

• They can be compared to experimentally-produced supercritical bedforms and field examples, allowing for a better understanding and reconstruction of paleo-flow conditions [2], [3], [4], [5].
• The sediments contain deformation features (disaggregation bands, see [1]). It is postulated that grain-size controls whether and how such bands can be generated. This benefits structural geologists.
• The sand can be used in analogue experiments, for instance, to generate synthetic disaggregation bands. These experiments need to be calibrated to the constituent size of the particles to determine the processes that take place.

## 1 Objective

A representative number of sand samples were taken from delta foreset beds of two delta lobes to classify grain-size distribution of the different bedforms present in the outcrop. It has been speculated in the literature that disaggregation bands only form in soft sediments of a certain grain-size, but this data shows the disaggregation bands formed in the poorest sorted, over a range of average grain-sizes, and in different types of sedimentary structures at this locality, suggesting grain-size (distribution) is not the most-important parameter. Furthermore, this outcrop contains deposits of supercritical flows, the grain-size distributions of which have been described only rarely in the literature until now.

## 2 Data Description

**Fig. 1:**
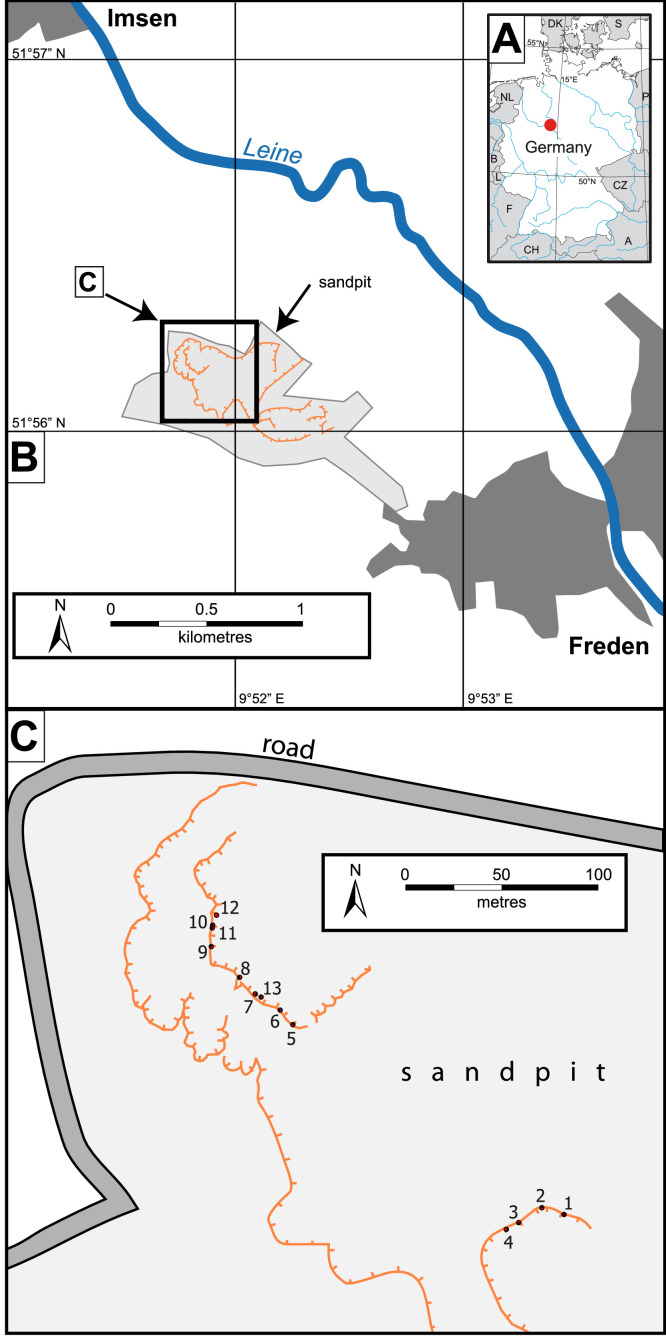
Location map. A) Location in Germany, B) Location of the sandpit between Freden and Imsen. C) Locations of the samples taken in the sandpit. Data from © GeoBasis-DE/BKG 2018.

**Fig. 2:**
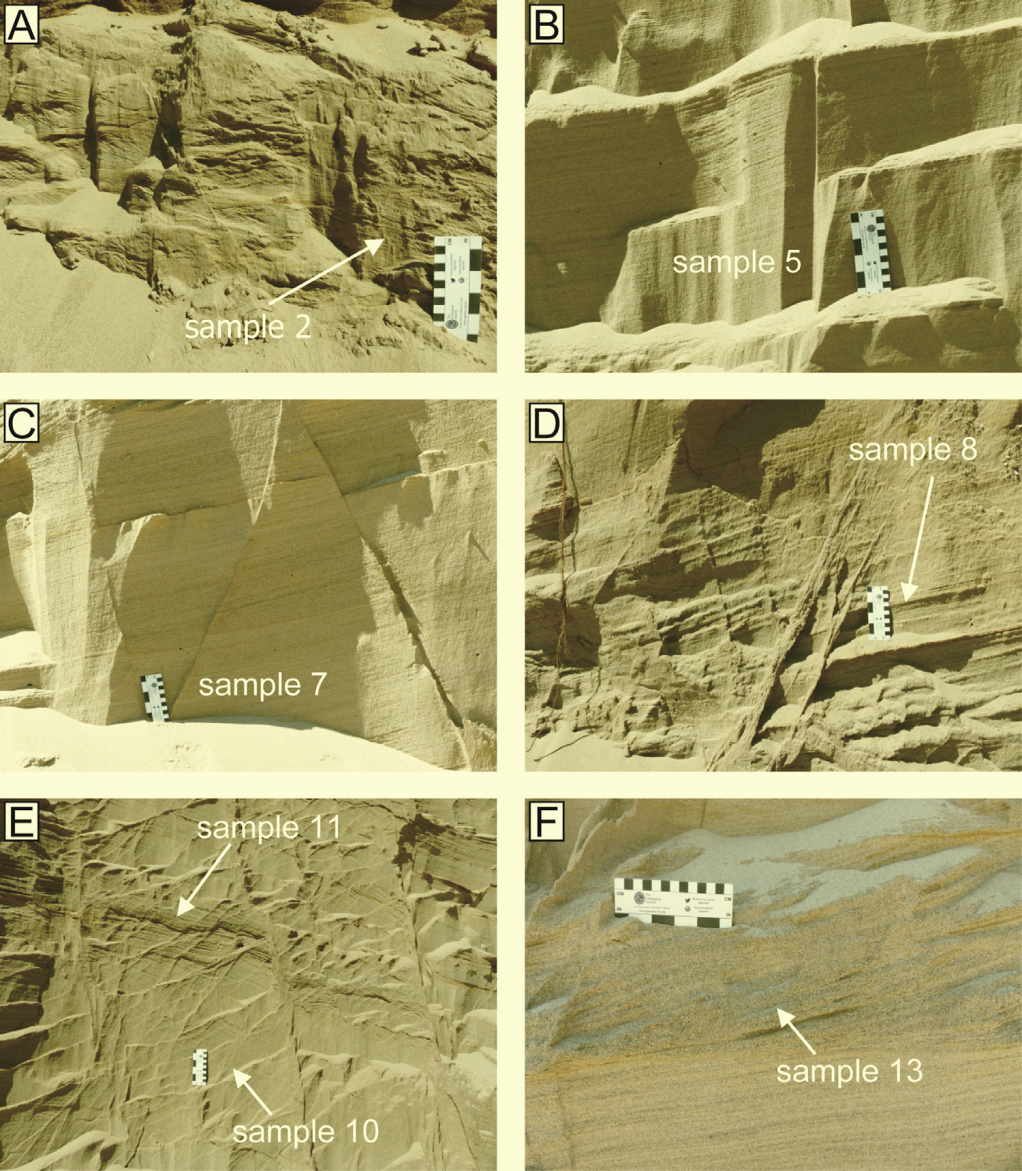
Examples of the bedforms from which the samples were taken. A) Deposits of 3D climbing ripples, B), C), D) and E) Deposits of stable antidunes. Note the disaggregation bands in D) to the left of the sample. F) Deposits of downstream-migrating antidunes. Scale is 13 cm wide and marked in centimetres and inches.

Table 1 shows the locations of samples (see also Fig. 1), the sedimentary bedforms (see Fig. 2), and whether disaggregation bands (deformation) were present at each sample location.

**Table 1:** Locations of samples and sedimentary structure of the samples. Datum WGS 84, UTM Zone 32U (EPSG Code 32632).

	Coordinates			
Sample	Geographic	UTM (easting northing)	Bedform	Deformation
1	N51°56′08.6′′ E9°51′50.8′′	559406 5754242	cR	Yes
2	N51°56′08.8′′ E9°51′50.3′′	559396 5754248	cR	Yes
3	N51°56′08.6′′ E9°51′49.6′′	559383 5754242	sAD	No
4	N51°56′08.5′′ E9°51′49.2′′	559376 5754238	sAD	No
5	N51°56′12.5′′ E9°51′44.5′′	559284 5754361	sAD	No
6	N51°56′12.8′′ E9°51′44.2′′	559279 5754370	sAD	No
7	N51°56′13.2′′ E9°51′43.7′′	559269 5754382	sAD	No
8	N51°56′13.5′′ E9°51′43.3′′	559261 5754391	sAD	No
9	N51°56′14.1′′ E9°51′42.7′′	559249 5754410	sAD	No
10	N51°56′14.4′′ E9°51′42.8′′	559251 5754419	sAD	Yes
11	N51°56′14.4′′ E9°51′42.8′′	559251 5754419	sAD	Yes
12	N51°56′14.6′′ E9°51′43.0′′	559251 5754425	sAD	No
13	N51°56′13.2′′ E9°51′43.9′′	559273 5754382	dAD	No

Abbreviations of bedforms: cR: 3D climbing ripples, sAD; stable antidunes, dAD: downstream-migrating antidunes. Deformation – yes means disaggregation bands were present in the outcrop.

Table 2 shows the grain-size measurements in micrometres. For valid laser diffraction, obscuration should be between 7-12%; for PIDS it should be between 30-80%.

**Table 2:** Grain-size statistics for the thirteen samples in micrometres.

Sample	mean	median	S.D.	coeff. v.	skew	kurtosis	obscur. (%)	PIDS (%)
1	210.03	207.33	97.86	46.59	0.17	0.60	10	71
2	177.75	179.05	91.39	51.41	0.25	0.63	10	74
3	376.01	334.15	218.71	58.17	1.65	4.06	9	74
4	286.46	277.96	118.17	41.25	0.14	0.42	9	71
5	289.05	286.62	116.03	40.14	-0.04	0.06	7	58
6	331.74	333.43	134.07	40.41	-0.18	0.04	7	66
7	342.19	312.86	200.25	58.52	1.89	5.99	7	65
8	331.16	329.89	129.03	38.96	-0.13	0.15	8	71
9	338.71	301.34	207.34	61.21	1.82	5.13	8	67
10	319.58	328.14	142.15	44.48	-0.26	-0.27	10	74
11	387.68	351.99	231.33	59.67	1.27	2.55	8	69
12	339.31	341.84	130.33	38.41	-0.27	0.27	7	68
13	452.91	429.68	248.00	54.76	0.67	0.91	9	74

Mean	321.74	308.79	158.82	48.77	0.54	1.58		

S.D.	71.43	63.43	54.16	8.79	0.83	2.13		

Abbreviations: S.D. – standard deviation, coeff. v. – coefficient of variance, obscur. – Obscuration, PIDS - Polarization intensity differential scattering obscuration.

Tables 2 and 3 show the average grain-size of the delta-foreset sediments to be 321 µm (1.78 ϕ), which is the range of medium sand, but they also show a range of sorting, from poor to moderately-well sorted, depending on the flow and depositional processes. With the exception of two examples (samples 7 and 9), the skew of the grain-size is low, below one, and both slightly negative and positive. Kurtosis of the grain-size curve also ranges greatly.

**Table 3:** Grain-size statistics for the thirteen samples in ϕ (ϕ = -log_2_(d), where d is the grain-size in mm, calculated from Table 2).

sample	mean	median	S.D.	sorting class.	skew	kurtosis
1	2.325	2.270	1.130	poor	0.406	2.971
2	2.618	2.482	1.263	poor	0.494	2.787
3	1.593	1.581	0.753	moderately	0.036	1.215
4	1.868	1.847	0.623	mod. well	0.160	1.235
5	1.858	1.803	0.638	mod. well	0.240	1.223
6	1.655	1.585	0.662	mod. well	0.292	1.282
7	1.717	1.676	0.753	moderately	0.173	1.307
8	1.651	1.600	0.605	mod. well	0.234	1.183
9	1.746	1.731	0.833	moderately	0.136	1.469
10	1.730	1.608	1.033	poor	0.478	2.103
11	1.541	1.506	1.232	poor	0.304	2.682
12	1.607	1.549	0.618	mod. well	0.284	1.337
13	1.293	1.219	1.361	poor	0.402	2.732

mean	1.78	1.73	0.89		0.28	1.81

S.D.	0.34	0.33	0.28		0.14	0.72

Abbreviations: S.D. – standard deviation, sorting class. - sorting classification according to [6].

**Fig. 3:**
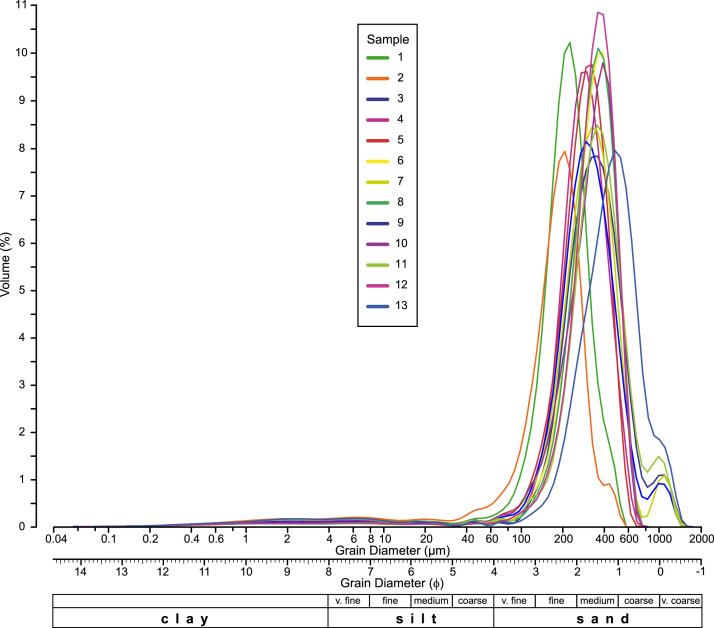
Logarithmic plot of grain-size against volume percent for all samples.

Fig. 3 shows the grain-size distributions for all thirteen samples. Clearly, all have a maximum at 200-400 µm (2.5-1 ϕ), as shown by the statistics, but, in addition, all the distributions have a long “fine tail” going down to 0.06 µm (14 ϕ). This fine fraction is separated from the sand by a data gap at 30-50 µm (5 ϕ) in most samples. This is an extra fine distribution that would not be seen in a classic sieve analysis, but the laser diffractometer is able to measure grain-size to 40 nm and is able thus to analyse this fine fraction. The amount of material under 40 µm is estimated to be 6-8% of the total volume. Another feature, apparent in some samples (samples 3, 7, 9, 11), is a distinct coarse fraction above 1 mm (0 ϕ).

**Fig. 4:**
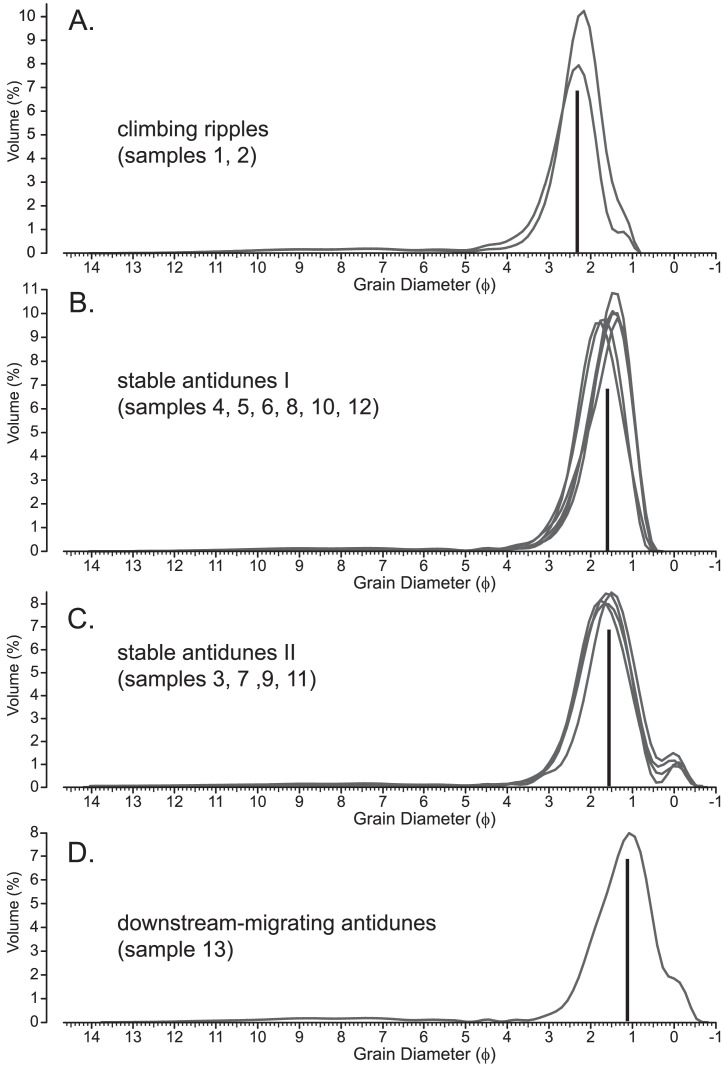
Logarithmic grain-size distribution for the different bedform types (from Table 1; stable antidunes are split in to two groups, because of the appearance of a separate coarse fraction in some samples (stable antidunes I and II). The bar represents the average median grain-size for all the curves in one plot.

Fig. 4 shows grain-size distribution plots according to the different bedforms. All curves have the characteristic fine fraction that indicates deposition of supension load by waning density flows on the lower delta slope. Samples 1 and 2 (Fig. 4A) are from climbing-ripple trough cross-laminated sands, deposited by subcritical density flows. They have the finest grain-size (mean 210 µm [2.325 ϕ]), compared to the other samples that were deposited by supercritical density flows. These sand fractions are also more symmetrical (skew 0.17) than other sediments. They have no coarser than sand fraction; the maximum grain-size is 600 µm.

Samples from deposits of stable aggrading antidunes can be split into two groups, based on whether they have a coarse fraction over 1 mm or not. Most of the stable antidune deposits have no coarse fraction (Fig. 4B). These sediments have an average mean grain-size of 330 µm (1.6 ϕ) and slightly negative or positive skew. The four samples from deposits of stable aggrading antidunes with a coarse fraction (Fig. 4C) have exact the same average mean grain-size as the group in Fig. 4B. However, they have in addition a distinct coarse fraction above 500 µm (0 ϕ). The curves of this group are very symmetrical, with positive skew and high kurtosis.

Sample 13 (Fig. 4D) was taken from deposits of downstream migrating antidunes, which have the coarsest mean grain-size (450 µm [1.2 ϕ]) as well as a stronger coarse fraction above 500 µm (0 ϕ). This causes the skew to become strongly positive.

This correlation of grain-size distribution with sedimentary bedform corresponds well to other field examples [6], and flume and tank experiments [3,7].

## 3 Experimental Design, Materials and Methods

Based on the work of [2] and [5], the Pleistocene Gilbert-delta deposits at Freden comprise three major bedforms, i.e., 3D climbing ripples, stable antidunes, and downstream-migrating antidunes. From each bedform, representative samples were taken, each sample was described in terms of sedimentology and structural characteristics. In this way, the thirteen samples represent a spectrum of possible grain-size distributions that occur at this site.

As described above, the grain-size distributions of the samples were then analysed using a laser diffractometer. As is standard for this kind of analysis, the samples were homogenised to ensure the measured sample of 1-2 g was representative. The software used to analyse the laser diffractometer data produced a grain-size distribution curve and basic statistics were carried out. For each sample, five grainsize profiles were run; if the individual values were within 1%, the curves were averaged. The data were exported as an Excel table. Further statistics (in Excel) were used to discriminate the various characteristics of the grain-size distributions.

## Ethics Statements

None.

## CRediT authorship contribution statement

**David C. Tanner:** Conceptualization, Methodology, Data curation, Writing – original draft, Visualization, Investigation, Writing – review & editing. **Christian Brandes:** Conceptualization, Methodology, Writing – review & editing. **Jutta Winsemann:** Conceptualization, Methodology, Writing – review & editing.

## Declaration of Competing Interest

The authors declare that they have no known competing financial interests or personal relationships that could have appeared to influence the work reported in this paper.

## Data Availability

Figure 1 (Original data) (Figshare). Figure 2 (Original data) (Figshare). Figure 3 (Original data) (Figshare). Figure 4 (Original data) (Figshare). Table giving grainsize distributions and statistics (Original data) (Figshare). Raw grainsize distribution curves for samples from Freden (Original data) (Figshare). Raw grainsize distribution curves as Excel tables (Original data) (Figshare). Raw grainsize distribution curves as Excel tables (Original data) (Figshare).
